# Examining chronic disease onset across varying age groups of Indian adults using competing risk analysis

**DOI:** 10.1038/s41598-023-32861-5

**Published:** 2023-04-10

**Authors:** Rashmi Rashmi, Sanjay K. Mohanty

**Affiliations:** Department of Population and Development, International Institute for Population Sciences, Mumbai, 400088 India

**Keywords:** Risk factors, Diseases, Epidemiology, Diagnosis, Geriatrics, Health policy, Public health

## Abstract

In low-and-middle-income countries, people develop chronic diseases at a younger age, leading to health-and-economic loss. Estimates of the age of onset of chronic disease provide evidence for policy intervention, but in the Indian context, evidence is limited. The present study aims to explore the onset of seven chronic diseases across adults and the elderly, along with the prognostic factors of chronic disease onset. Using Wave 1 data of the Longitudinal Ageing Study in India (LASI), we estimated the statistical distributions, the median age at onset, and Loglogistic and Weibull accelerated failure time model to understand the onset of seven medically diagnosed self-reported chronic diseases across age groups. We also obtained the sub-distribution hazard ratio (SHR) from the Fine-Gray model to determine the risk of contracting selected chronic diseases in a competing risk setup. The seven chronic diseases– hypertension, diabetes, lung disease, heart disease/stroke, arthritis, neurological disease, and cancer– were developing early, especially in individuals aged 45–54 and 55–64. Arthritis risk was higher in rural areas, and physically active adults and elderly were 1.32 times (95% CI 1.12–1.56) more likely to develop heart disease/stroke. The emerging evidence of the early onset of neurological diseases in middle-aged adults (i.e., among the 45–54 age group) reminds us of the need to reinforce a balance between the physical and mental life of individuals. The early onset of chronic diseases in the independent and working-age category (45–54 years) can have many social and economic implications. For instance, it can create a greater healthcare burden when these individuals grow older with these diseases. Further, disease-specific interventions would be helpful in reducing future chronic disease burden.

## Introduction

Increased human longevity is associated with increased risks of non-communicable diseases (NCDs)^1–3^. Today, over 77% (31.4 million) of all NCD deaths occur in low- and middle-income countries (LMICs)^4^. While population aging had fueled chronic disease deaths among the elderly worldwide, the early onset of chronic conditions in LMICs affects both the working and the elderly population^5,6^. For instance, an estimated 15 million people die annually due to NCDs in the working age group of 30 to 69 years, among whom 85% are from LMICs^4^. While evidence from a few developed countries shows that morbidity is being compressed into a shorter period, many developing countries are undergoing morbidity expansion^7,8^.

With almost one-fifth of the world’s population, India is also undergoing a major health and epidemiological shift^9^. For instance, while the share of communicable diseases decreased from 61% in 1990 to 33% in 2016, there was a corresponding increase in the years lost due to premature death and chronic disease from 30% in 1990 to 55% in 2016^9^. In 2016, 63% of all deaths in India were due to chronic diseases, among which cardiovascular diseases were the most significant contributor (27%), followed by chronic respiratory diseases (11%), cancer (9%), diabetes (3%) and other NCDs (13%)^10^. Recent evidence suggests that 56.5% of the total disease burden in India is attributable to NCDs, and about 28% of older adults have multiple diseases^11,12^. This aligns with the morbidity expansion hypothesis; however, the continuous increase in chronic disease prevalence in every age group challenges the best-fit morbidity theory, especially in developing countries^13^. For instance, an Indian study found that many NCDs have developed at a younger age in recent decades^14^. Another study reported an earlier occurrence of coronary heart disease in an individual’s life^15^. Such evidence turns our attention to the importance of the age at which an individual first contracts a disease to adequately understand future healthcare needs and spending from a policy perspective. In this context, the present paper examined the age of onset of seven chronic diseases and the associated risk factors using the latest nationally-representative data of middle-aged and older adults in India.

The rationale for the present study is as follows: First, the estimates of the age of onset of NCDs remain unclear in India. Estimates of the age of onset of the first chronic disease provide an in-depth view of the relationship between life expectancy and health status and allow for a proper explanation of future healthcare needs and spending from a policy perspective^16^. Secondly, while there is extensive evidence on the risk factors of non-communicable diseases in India, research showing the prognostic factors predicting the onset of chronic diseases is limited^2,14,17,18^. The present study takes advantage of the availability of comprehensive information on individuals in the first wave of the Longitudinal Ageing Study to explore the individual, behavioural and socio-demographic factors of chronic disease onset. Thirdly, only one Indian study has so far found that the age of onset is lower and distinct for most NCDs and that sex and educational attainment have a role in the early onset of NCDs^14^. However, these conclusions were based on limited information on risk factors, and the age of onset of the disease was derived indirectly.

In addition, the hazard function in the literature provides only the instantaneous risk of getting a particular chronic disease, ignoring the fact that an individual might have already been affected by other chronic diseases by that age. From a methodological point of view, there is a need to consider the age at which an individual is exposed to a specific chronic disease for the first time, keeping the risk of the rest of the chronic diseases as a competing risk. So, the present study aims to explore the onset of specific chronic diseases and their association with different characteristics of adults in competing risk setups.

## Method

### Data

The present study used the baseline (Wave 1) data from the Longitudinal Ageing Study in India (LASI) conducted from April 2017 to December 2018, using the multistage, stratified, cluster sampling design. A total of 73,396 individuals aged 45 years and above and their spouses (irrespective of age) from all the states and union territories of India were interviewed. The response rates at household and individual levels were 95.8% and 87.5%, respectively^19^. LASI Wave 1 collected detailed demographic, health, economic, social, and psychological information of the eligible members and in-depth information on the burden of diseases, functional health, healthcare, and social and economic well-being. The sample design, survey instruments, and preliminary findings are available in the survey report^19^. All the experimental protocols were approved by the Indian Council of Medical Research (ICMR) Ethics Committee and written informed consent was obtained from all subjects and/or their legal guardian(s)^19^. All methods were carried out in accordance with relevant guidelines and regulations.

After excluding the missing observations from the selected variables, the final analytical sample was 65,258 individuals aged 45 years and above in India.

### Outcome variable

We used seven medically diagnosed self-reported chronic diseases such as hypertension, diabetes, lung disease, heart disease or stroke, arthritis, neurological diseases, and cancer. The main outcome variable was the age of onset of these chronic diseases (in years) among individuals diagnosed by medical health professionals. The age of disease onset was derived from a direct question in the survey: “When were you first diagnosed (by health professionals) with the specific chronic disease in years or age?”.

### Explanatory variables

The present study uses three age groups of adults and elderly, namely “45–54 years”, “55–64 years,” and “65 + years” as the main explanatory variable. Based on the availability of information in the survey, the association of age of onset (and risk) of different chronic diseases across varying age groups was assessed, controlling individual, behavioural and socio-demographic characteristics.

The individual factors were sex (male, female), educational attainment (none, less than 5 years, 5–9 years, 10 years or more), working status (currently working, ever worked but not working, never worked), marital status (married, widowed/others), living arrangement (living alone; living with spouse, children, and/or others; living with children and/or others), and depression (no, yes). The behavioural characteristics included tobacco consumption (never, past user, current user), alcohol consumption (never, past user, current user), and physical activity (inactive, active). The socio-demographic characteristics included caste (scheduled caste[SC]/scheduled tribe[ST], other backward class[OBC], others), religion (Hindu, Muslim, Other), residence (rural, urban), and monthly per capita expenditure (MPCE) quintile of household (poorest, poorer, middle, richer, richest). A description of the variables is provided in Appendix 1.

### Statistical analysis

We estimated the statistical distributions of the age of onset of seven chronic diseases using mean, standard deviation, median, and interquartile range across age groups. Kernel density plots were drawn to visualize the distribution of different chronic diseases across the age groups. A kernel density plot provides a smoother distribution of variables over a continuous interval or time period^20^. In our study, the peaks depict the age where the chronic diseases are concentrated over the given interval.

Note that the onset data contains right-censored observations, i.e., those individuals who did not develop any disease till the time of the survey. Thus, the median age of onset of developing any chronic disease across the age group categories was calculated in the bivariate analysis^21^. The median age of onset (or median survival time in survival analysis) describes the age at which half of the population experiences a particular chronic disease in the present analysis. The log-rank test was used to test the difference in the probability of an event (onset of any disease) between the populations (age group categories).

An accelerated failure time (AFT) model (a parametric survival regression method) was used to measure the acceleration or deceleration of survival time across the age groups^21^. The parametric survival regression model allows us to choose the underlying distribution of time-to-event, accounting for the censored observations in the data. Moreover, the present study uses statistical measures like Alkaline Information Criterion (AIC) and Bayesian Information Criterion (BIC) to check the survival model fitness^21^.

AFT assumes that covariates act multiplicatively on failure time and follow log time parametrization. This provides an exponential regression coefficient called AFT’s time ratio (TR). If TR = 1, then time passes at its normal rate. If TR > 1, then the expected time increases and failure is expected to occur later. If TR < 1, the expected time decreases and failure is expected to occur sooner.

In survival analysis, sometimes other events may preclude the time of occurrence of the primary event of interest. Such events are denoted as competing risks. For instance, in this study, the first occurrence of a specific disease (primary outcome) may have been affected by the prior existence of other chronic diseases by that age. Thus, there is a need to consider such competing risks that prevent an event of interest, especially while studying different chronic diseases. In a competing risk setup, under each cause of event occurrence, a hazard function is considered under the covariate’s presence. The number of failures from causes other than the cause of interest reduces the actual number of failures from the cause of interest and influences the probability of failure^22^. In accordance, Fine and Gray introduced the cumulative incidence function (CIF)-based proportional hazard model (in sub-distribution hazard function) to analyse competing risks in survival data^23^. In this model, for a covariate x_i_, the sub-distribution hazard ratio (SHR) for the cause j (j = 1, …, p) is given by exp(β_ji_), keeping all the covariates at a fixed level, where β_ji_ is the regression coefficient. Thus, the multivariate association between specific chronic diseases and different covariates was presented using a sub-hazard ratio along with p-value at 5% level of significance and confidence interval.

Further, the variance inflation factor (VIF) test shows no evidence of collinearity among the independent variables in the present study^24^. All the analyses were carried out in the STATA 14 software^25^. We used “syvset” command to account for the survey’s complex nature and for the generalizability of the findings^21^.

## Results

Table 1 shows the individual, behavioural and socio-demographic characteristics of 65,258 individuals aged 45 years and above in India. Adults and elderly were divided into three different age groups such as 23,005 were in the 45–54 age group, 19,631 in the 55–64 age group, and 22,622 individuals aged 65 years and above. Among all adults and elderly, nearly 54.24% were females, and 45.76% were males. About 50.6% of adults and elderly had never received any education, and only 17.95% had received ten years or more of education. Almost one in two adults was currently working, and 74.1% of adults were married or in live-in relationships. Living with a spouse, children, and others was common (72.62%) among the adults and elderly; in contrast, only 3.69% of individuals aged 45 + were living alone. Almost 8.33% of adults and elderly faced depression in the total population. About 31.42% of individuals consumed tobacco, 6.22% consumed alcohol, and 11.64% were physically inactive. We observed that about 42% of adults and elderly belonged to the poorest/poor wealth quintile households, and 68.71% resided in rural communities. Most individuals belonged to the OBC caste (45.51%) and Hindu religion (82.2%).

**Table 1:** Percent distribution of adults and elderly by individual, behavioral and socio-demographic characteristics in India, LASI (2017–18).

Characteristics	N	%
Individual		
Age groups		
45–54	23,005	35.25
55–64	19,631	30.08
65 +	22,622	34.67
Sex		
Male	29,862	45.76
Female	35,396	54.24
Level of education		
None	33,018	50.6
Less than 5 years	7,140	10.94
5–9 years completed	13,385	20.51
10 years or more	11,715	17.95
Working status		
Currently working	30,470	46.69
Ever worked but not working currently	17,787	27.26
Never worked	17,002	26.05
Marital status		
Currently married/live in	48,359	74.1
Separated/divorced/widowed/never	16,899	25.9
Living arrangement		
Living alone	2409	3.69
Living with spouse, children and/or others	47,393	72.62
Living with children and/or others	15,456	23.68
CIDI-SF depression status		
Not depressed	59,819	91.67
Depressed	5,439	8.33
Behavioural		
Tobacco consumption		
Never used	40,987	62.81
Past user	3769	5.77
User	20,502	31.42
Alcohol consumption		
Never used	55,380	84.86
Past user	5816	8.91
Current user	4061	6.22
Physical activity status		
Physically inactive	7597	11.64
Physically active	57,661	88.36
Socio-demographic		
Caste		
SC/ST	18,147	27.81
OBC	29,696	45.51
Others	17,415	26.69
Religion		
Hindu	53,641	82.2
Muslim	7335	11.24
Others	4281	6.56
Place of residence		
Rural	44,838	68.71
Urban	20,420	31.29
MPCE quintile		
Poorest	13,650	20.92
Poorer	13,840	21.21
Middle	13,288	20.36
Richer	12,731	19.51
Richest	11,749	18
Total	65,258	100

We estimated the prevalence of at least one chronic disease to be 41.73% (95% CI: 40.59 to 42.87) among adults and the elderly in India (Table 2). Among the seven selected diseases, self-reported diagnosed hypertension had the highest prevalence (26.82%), followed by diabetes (12.24%), arthritis (9.01%), lung disease (6.43%), heart disease or stroke (5.34%), neurological diseases (2.17%), and cancer (0.63%).

**Table 2:** Age sex-adjusted prevalence of self-reported diagnosed chronic diseases among adults and elderly in India, LASI (2017-18).

Sr No	Chronic diseases	Sample size (N)	Adjusted prevalence	95% CI
1	Hypertension	65,239	26.82	[25.72, 27.92]
2	Diabetes	65,236	12.24	[11.15, 13.34]
3	Lung disease	65,246	6.43	[5.75, 7.12]
4	Heart disease/stroke	65,245	5.34	[4.88, 5.80]
5	Arthritis	65,247	9.01	[7.93, 10.09]
6	Neurological	65,238	2.17	[1.89, 2.45]
7	Cancer	65,245	0.63	[0.52, 0.75]
Total	Any chronic disease	65,223	41.73	[40.59, 42.87]

Adjusted for age and sex; after excluding missing observations these sample sizes were considered for analysis of respective chronic diseases.

Table 3 shows the age of onset (in years) of chronic diseases across varying age groups of adults and elderly in India. We found that the mean and median age of onset of any chronic disease was 53.6 years and 53 years, respectively. The median age of onset of any chronic diseases was around 44 to 45 years in the 45–54 age group individuals, followed by 52 to 54 years in individuals of the 55–64 age group, and 62 to 65 years in the 65 + age group individuals. A similar trend was observed in the bivariate association of the median age of onset of any chronic disease with individual, behavioural and socio-demographic characteristics across age groups (Appendix 2). While diabetes and cancer show lower mean age at onset among the 65 + age group population, the lower mean age for lung disease onset was prevalent in the 45–54- and 55–64-age groups (Table 3). Further, the lower median age at onset for heart disease/stroke and neurological disease in middle-aged adults (45–54 age group) indicates the life-threatening risks to the working-age population of India.

**Table 3:** Statistical distribution of age of onset of chronic diseases among adults and elderly in India, LASI (2017-18).

Chronic diseases	Age groups of adults and elderly											
	45–54			55–64			65 +			All		
	Mean (SD)	Median	IQR	Mean (SD)	Median	IQR	Mean (SD)	Median	IQR	Mean (SD)	Median	IQR
Hypertension	44.0 (6.3)	45	41–48	53.1 (6.6)	54	50–58	63.1 (10.6)	64	58–70	55.0 (11.6)	55	47–63
Diabetes	44.1 (6.4)	45	42–49	52.5 (7.4)	54	50–57	61.1 (11.2)	62	57–68	53.8 (11.3)	54	46–61
Lung disease	41.8 (9.0)	44	38–48	47.3 (12.6)	52	40–57	62.7 (13.5)	64	58–70	53.7 (15.4)	55	45–64
Heart disease/stroke	43.4 (6.6)	44	42–48	53.2 (7.0)	55	50–58	65.4 (8.7)	65	61–70	57.8 (11.7)	59	50–65
Arthritis	44.4 (6.1)	45	42–48	53.5 (6.0)	54	51–57	64.0 (8.9)	64	60–69	55.9 (10.9)	56	49–63
Neurological	41.6 (8.5)	44	40–48	52.0 (9.2)	54	50–58	65.6 (11.8)	66	61–72	54.0 (14.4)	54	45–64
Cancer	44.3 (6.8)	45	42–49	51.2 (9.0)	54	46–58	62.1 (10.7)	63	54–68	53.3 (11.8)	51	46–61
Any chronic disease	43.3 (6.9)	45	41–48	51.4 (8.6)	54	48–57	62.1 (11.5)	64	57–69	53.6 (12.3)	53	45–62

*IQR* inter quartile range = P25 and P75.

**Figure 1:**
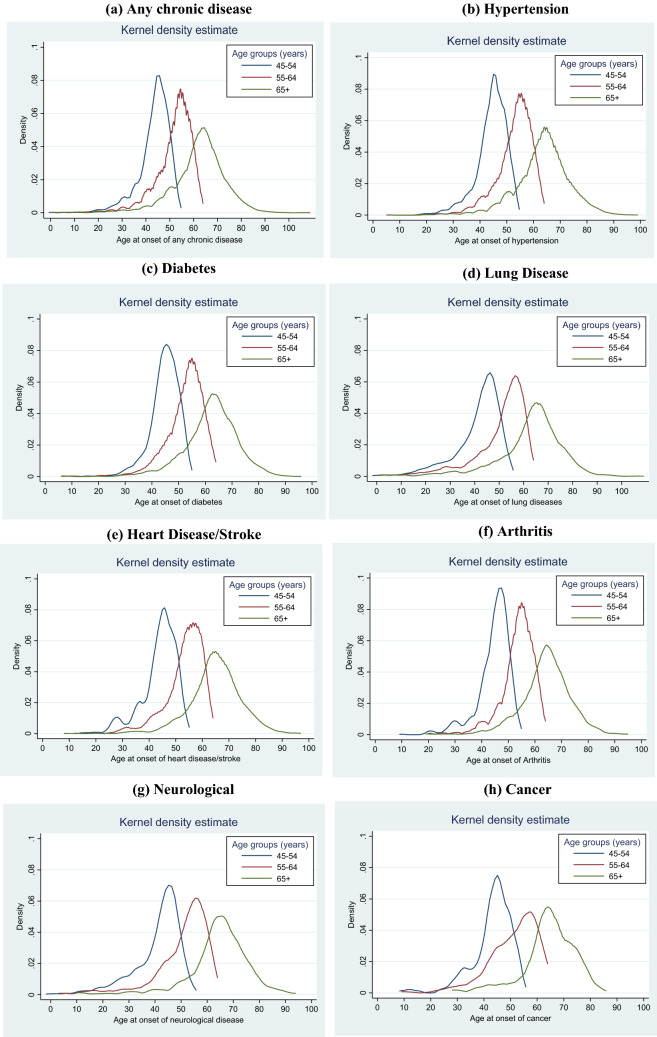
Kernel density estimation of age of onset of any and selected chronic diseases across age groups of adults and elderly in India, LASI (2017-18). (**a**) Any (**b**) Hypertension (**c**) Diabetes (**d**) Lung disease (**e**) Heart disease/Stroke (**f**) Arthritis (**g**) Neurological (**h**) Cancer.

The Kernel density plot provides a visual representation of the distribution of chronic diseases across the age groups of adults and the elderly (Fig. 1). The individuals in the 45–54 age group were likely to be diagnosed with a chronic disease earlier than those in the 65 + age group population. For instance, the middle-aged adults in the 45–54 age group showed the earliest peak or concentration of chronic diseases (namely hypertension, diabetes, lung disease, heart disease or stroke, arthritis, neurological diseases, and cancer) at the age of around 42–45 years, followed by 55–64 age group individuals (peak at around 55 years), and those in 65 + age group (peak at around 65–70 years) (Fig. 1a–h). The highest peak in cancer among middle-aged adults (45–54 age group) emerged at 45 years of age; however, around 30–35 years of age, a small peak can be observed, indicating the cancer risk in initial ages. Such a pattern was also observed in the onset of heart disease/stroke.

To select the best fit model for parametric survival regression, we compared the Alkaline Information Criterion (AIC) and Bayesian Information Criterion (BIC) scores of four prominent survival regression models (Exponential, Weibull, Lognormal, and Loglogistic) across all seven selected chronic diseases^21^. The model with lower AIC and BIC values denotes a better fit for accelerated failure time regression across diseases. Based on the results, Loglogistic accelerated failure time model was best fit for hypertension, diabetes, heart disease/stroke, arthritis, and cancer, and the Weibull accelerated failure time model was employed in lung and neurological diseases (Appendix 3).

The Loglogistic and Weibull accelerated failure time model provides the adjusted association of individual, behavioural and socio-demographic characteristics with the onset of chronic diseases among individuals (Appendix 4). Results of the main explanatory variable, i.e., age groups of adults and elderly, were presented in Table 4, showing clear evidence of the early onset of chronic diseases in 45–54- and 55–54-years age group individuals compared to those in the 65 + age group population. In the case of hypertension, survival time decreased from 0.86 (95% CI 0.85–0.88) in 55–64 years age group individuals to 0.76 (95% CI 0.74–0.79) in 45–54 age group adults. Lesser survival time represents the earlier occurrence of disease by a covariate. For instance, individuals in the 45–54- and 55–54-years age groups were 23% and 15% more likely to experience diabetes, respectively, than the 65 years and above age group individuals. Notably, we found that the relationship between age groups and onset of lung disease, heart disease/stroke, arthritis, neurological diseases, and cancer was similar to that of hypertension and diabetes, suggesting the earlier occurrence of all chronic diseases in the 45–54 age group. Age of onset for hypertension and cancer was earlier for females than for males (Appendix 4). Depression was highly related to the early onset of hypertension, heart disease/stroke, arthritis, and neurological diseases. Surprisingly, heart disease/stroke onset was common among physically active individuals. Residing in an urban setting and belonging to the richest wealth quintile household accelerates the onset of almost all chronic diseases.

**Table 4:** Accelerated failure time from Log-logistic and Weibull regression model showing the association of age groups of adults and elderly with selected chronic diseases in India, LASI (2017-18).

Characteristics	Chronic diseases													
	Hypertension		Diabetes		Lung disease		Heart disease & stroke		Arthritis		Neurological		Cancer	
	TR	Confidence Interval	TR	Confidence Interval	TR	Confidence Interval	TR	Confidence Interval	TR	Confidence Interval	TR	Confidence Interval	TR	Confidence Interval
Age groups														
65 + (Ref)														
55–64	0.86**	(0.85–0.88)	0.85**	(0.82–0.87)	0.85**	(0.82–0.89)	0.86**	(0.83–0.89)	0.86**	(0.84–0.88)	0.83**	(0.8–0.87)	0.81**	(0.76–0.87)
45–54	0.76**	(0.74–0.79)	0.77**	(0.73–0.83)	0.82**	(0.74–0.92)	0.78**	(0.74–0.83)	0.77**	(0.73–0.81)	0.68**	(0.64–0.72)	0.7**	(0.64–0.77)

Adjusted for individual, behavioral and socio-demographic characteristics; state fixed effect is also considered; ** p < 0.05; TR = time ratio; Loglogistic accelerated failure time model was fitted for hypertension, diabetes, heart disease/stroke, arthritis, and cancer and the Weibull accelerated failure time model was employed in lung and neurological diseases.

Table 5 shows the sub-hazard ratio for the competing risk setup of seven chronic diseases among adults and the elderly in India. Even after adjusting for individual, behavioural, and socio-demographic characteristics, risks across middle-aged adults remained significantly high for all seven chronic diseases. For instance, compared to the individuals in the 65 years and above age group, the hypertension risk was higher among 45–54 years age group individuals (SHR: 2.08; 95% CI 1.98–2.18). The risk of getting a neurological disease was 2.47 times (95% CI 2.04–2.99) higher in the 45–54 age group than those in the 65 + age group. The risk of developing hypertension (SHR: 1.49; 95% CI 1.41–1.56), arthritis (SHR: 1.64; 95% CI 1.49–1.8), and cancer (SHR: 1.85; 95% CI 1.31–2.61) was higher among females. On the contrary, diabetes, lung disease, heart disease/stroke, and neurological disease were more prominent among male adults and the elderly. The risk of hypertension and diabetes onset was 1.37 times (95% CI 1.3–1.46) and 1.65 times (95% CI 1.52–1.8) higher among adults and elderly with ten years or more of education compared to those who were illiterate. Compared to the currently employed adults and elderly, the risk of developing any chronic diseases except arthritis and cancer was higher among individuals who had never worked or those who had worked but were currently unemployed. Adults and elderly living alone were at lesser risk of developing heart disease/stroke than those living with spouses, children, and others. Although depression was a prominent predictor of developing many chronic diseases, the risk of neurological disease onset due to depression was significantly higher than the other chronic conditions (SHR: 2.81; 95% CI 2.32–3.4). Considering the behavioural characteristics, being a past tobacco user doubles the risk of getting cancer (SHR: 2.51; 95% CI 1.68–3.74) and shows 1.7 times (95% CI 1.49–1.94) and 1.59 times (95% CI 1.36–1.85) higher likely to develop lung and heart disease/stroke respectively. Physically active adults and the elderly were 1.32 times (95% CI 1.12–1.56) more likely to develop heart disease/stroke. Compared to the SC/ST population, the risk of chronic disease onset was higher in the OBC and other caste categories. Compared to the poorest wealth quintile households, the hazard risks for hypertension, diabetes, lung disease, heart disease/stroke, and cancer were highest among the adults and elderly in the richest wealth quintile households. Adults in urban areas were also more likely to develop hypertension, diabetes, and heart disease/stroke.

**Table 5:** Sub-distribution hazard ratio (SHR) obtained from the Fine-Gray models for true risk of getting selected chronic diseases (competing risks) with individual, behavioral and socio-demographic characteristics of adults and elderly in India, LASI (2017-18).

Characteristics	Chronic diseases													
	Hypertension		Diabetes		Lung disease		Heart disease & stroke		Arthritis		Neurological		Cancer	
	SHR	Confidence Interval	SHR	Confidence Interval	SHR	Confidence Interval	SHR	Confidence Interval	SHR	Confidence Interval	SHR	Confidence Interval	SHR	Confidence Interval
Individual														
Age groups														
65 + (Ref)														
55–64	1.37**	(1.31–1.43)	1.4**	(1.32–1.5)	1.2**	(1.09–1.31)	1.23**	(1.1–1.37)	1.32**	(1.22–1.43)	1.53**	(1.28–1.84)	2.07**	(1.58–2.7)
45–54	2.08**	(1.98–2.18)	1.66**	(1.55–1.79)	1.49**	(1.34–1.65)	1.34**	(1.17–1.54)	1.83**	(1.68–1.99)	2.47**	(2.04–2.99)	2.38**	(1.76–3.21)
Individual's sex														
Male (Ref)														
Female	1.49**	(1.41–1.56)	0.79**	(0.74–0.86)	0.75**	(0.67–0.83)	0.53**	(0.47–0.61)	1.64**	(1.49–1.8)	0.77**	(0.63–0.95)	1.85**	(1.31–2.61)
Level of education														
None (Ref)														
Less than 5 years	1.18**	(1.11–1.25)	1.28**	(1.16–1.4)	1.01	(0.89–1.15)	1	(0.85–1.17)	1.08	(0.97–1.2)	0.94	(0.74–1.2)	1.43	(1–2.03)
5–9 years completed	1.25**	(1.18–1.31)	1.4**	(1.3–1.51)	0.96	(0.86–1.07)	1.13	(0.99–1.28)	0.95	(0.86–1.05)	1.1	(0.91–1.34)	1.13	(0.82–1.55)
10 years or more	1.37**	(1.3–1.46)	1.65**	(1.52–1.8)	0.67**	(0.58–0.78)	1.04	(0.9–1.21)	0.83**	(0.74–0.93)	0.89	(0.7–1.13)	0.97	(0.66–1.43)
Working status														
Currently working (Ref)														
Ever worked but not working currently	1.11**	(1.05–1.16)	1.25**	(1.16–1.34)	1.47**	(1.33–1.62)	1.86**	(1.64–2.11)	1.09	(1–1.18)	1.53**	(1.26–1.86)	1.53**	(1.14–2.05)
Never Worked	1.07**	(1.02–1.13)	1.38**	(1.27–1.5)	1.21**	(1.07–1.38)	1.64**	(1.4–1.91)	0.88**	(0.8–0.97)	1.53**	(1.22–1.92)	1.31	(0.93–1.84)
Marital status														
Currently married/live in (Ref)														
Separated/divorced/widowed/never	0.99	(0.86–1.13)	0.9	(0.75–1.08)	1.33	(0.94–1.87)	1.47	(0.94–2.32)	0.84	(0.67–1.06)	1.12	(0.65–1.91)	0.97	(0.38–2.46)
Living arrangement														
Living alone (Ref)														
Living with spouse, children and/or others	0.96	(0.82–1.13)	1.15	(0.92–1.44)	1.49**	(1.01–2.19)	2.03**	(1.21–3.42)	1.08	(0.83–1.41)	0.96	(0.51–1.79)	1.7	(0.45–6.39)
Living with children and/or others	1.06	(0.96–1.16)	1.04	(0.89–1.21)	1.16	(0.93–1.44)	1.18	(0.88–1.58)	1.08	(0.91–1.28)	0.92	(0.65–1.32)	1.42	(0.68–2.95)
CIDI-SF depression status														
Not depressed (Ref)														
Depressed	1.09**	(1.02–1.17)	1.04	(0.93–1.16)	1.07	(0.93–1.23)	1.67**	(1.44–1.93)	1.33**	(1.18–1.49)	2.81**	(2.32–3.4)	1.53**	(1.06–2.19)
Behavioural														
Tobacco consumption														
Never used (Ref)														
Past user	0.95	(0.88–1.03)	0.91	(0.81–1.02)	1.7**	(1.49–1.94)	1.59**	(1.36–1.85)	0.93	(0.8–1.08)	1.29	(0.96–1.73)	2.51**	(1.68–3.74)
User	0.89**	(0.85–0.93)	0.79**	(0.73–0.85)	1.15**	(1.04–1.26)	0.92	(0.81–1.04)	1.01	(0.92–1.09)	1.37**	(1.14–1.64)	1.12	(0.82–1.52)
Alcohol consumption														
Never used (Ref)														
Past user	1	(0.94–1.08)	1.02	(0.92–1.13)	1.11	(0.98–1.27)	1.1	(0.95–1.28)	1.13	(1–1.28)	1.12	(0.88–1.43)	1.25	(0.85–1.84)
Current user	0.92	(0.84–1)	0.83**	(0.73–0.94)	0.96	(0.81–1.14)	0.81**	(0.65–1)	1	(0.87–1.16)	0.83	(0.63–1.09)	0.4**	(0.18–0.87)
Physical activity status														
Physically inactive (Ref)														
Physically active	0.93**	(0.88–0.99)	0.99	(0.91–1.08)	0.94	(0.83–1.07)	1.32**	(1.12–1.56)	1.06	(0.95–1.19)	1.04	(0.83–1.3)	1.41	(0.92–2.16)
Socio-demographic														
Caste														
SC/ST (Ref)														
OBC	1.05	(0.99–1.1)	1.14**	(1.05–1.23)	1.05	(0.95–1.16)	1	(0.88–1.14)	1.14**	(1.04–1.25)	1.3**	(1.06––1.59)	1.06	(0.76–1.49)
Others	1.07**	(1.02–1.13)	1.22**	(1.12–1.33)	0.95	(0.85–1.07)	0.99	(0.87–1.14)	1.04	(0.94–1.15)	1.15	(0.92–1.42)	1.21	(0.87–1.68)
Religion														
Hindu (Ref)														
Muslim														
Others	1.13**	(1.06–1.2)	1.25**	(1.14–1.37)	1.18**	(1.04–1.34)	1.3**	(1.12–1.51)	1.04	(0.93–1.17)	0.78	(0.61–1)	1.2	(0.83–1.74)
Place of residence	1.09**	(1.02–1.16)	1.18**	(1.07–1.29)	0.99	(0.84–1.18)	1	(0.84–1.2)	0.85**	(0.75–0.98)	0.92	(0.67–1.26)	1.13	(0.74–1.73)
Rural (Ref)														
Urban	1.39**	(1.34–1.45)	1.73**	(1.62–1.84)	0.9**	(0.82–1)	1.19**	(1.07–1.33)	0.78**	(0.72–0.84)	0.88	(0.74–1.05)	1.05	(0.8–1.36)
MPCE quintile														
Poorest (Ref)														
Poorer	1.11**	(1.04–1.18)	1.13**	(1.03–1.25)	1.17**	(1.04–1.33)	1.07	(0.91–1.25)	1	(0.9–1.12)	1.1	(0.87–1.4)	1.06	(0.71–1.57)
Middle	1.21**	(1.14–1.28)	1.27**	(1.16–1.4)	1.2**	(1.06–1.36)	1.19**	(1.02–1.39)	0.97	(0.87–1.08)	1.18	(0.93–1.49)	1.06	(0.71–1.59)
Richer	1.28**	(1.2–1.35)	1.43**	(1.3–1.56)	1.21**	(1.06–1.38)	1.16	(0.99–1.36)	1.02	(0.92–1.14)	1.16	(0.91–1.47)	1.41	(0.97–2.05)
Richest	1.3**	(1.23–1.39)	1.59**	(1.45–1.75)	1.47**	(1.28–1.67)	1.37**	(1.17–1.61)	0.99	(0.88–1.1)	1.18	(0.92–1.51)	1.82**	(1.25–2.65)

State fixed effect is also considered.

*SHR* sub-hazard ratio.

**p < 0.05.

## Discussion

The epidemiological shift from communicable to non-communicable diseases has the potential threat of increasing the burden of diseases among adults and the elderly. The shrinking of healthy years due to the early onset of diseases, especially among middle-aged adults, has raised the alarm for the double expansion of chronic diseases (living with a chronic condition from early life till death) in India. The present study revealed many important findings to understand the onset of chronic diseases across the varying age groups of adults and elderly in India, along with the prognostic factors leading to such risks. First, using large-scale data of adults and the elderly, we found that seven chronic diseases, namely, hypertension, diabetes, lung disease, heart disease/stroke, arthritis, neurological disease, and cancer, are developing at an early stage of life. For instance, the median age of onset of any chronic disease was 64 years in the 65 + age group individuals compared to 45 years in middle-aged adults (in the 45–54 age group). After adjusting the covariates, the results of the Loglogistic and Weibull accelerated failure time model confirmed the lower survival of, or the early onset of all diseases in, the 45–54 age group individuals. Second, the age group differences in the median age of onset of any chronic disease persisted for the individual, behavioural and socio-demographic characteristics. However, the wider gap in the median age at disease onset was observed across different characteristics of the elderly. For instance, while the age at onset of any disease in the rural 65 + age group was 66 years, almost half of the elderly (65 + age group) residing in urban areas develop a chronic disease at the age of 63. Third, the early occurrence of neurological disease in the 45–54 and 55–64 age groups suggests the growing need to offer neurological services in the form of awareness, identification, treatment, and rehabilitation, consistent with a prior study^26^. Early onset of hypertension, arthritis, and cancer was evident in females, whereas diabetes, lung disease, heart disease/stroke, and neurological disease were more prominent among male adults and the elderly. The risk of developing chronic diseases was significantly higher among urban residents and those in the rich wealth quintile households.

Fourth, the competing risk setup confirmed the higher risk of all chronic diseases, especially hypertension and neurological disease, in individuals aged 45–54 and 55–64. Higher education was associated with a greater risk of hypertension and diabetes among adults and the elderly. This may be explained by the fact that higher education brings more prosperity and that wealthier people are more likely to adopt an unhealthy lifestyle, which may trigger lifestyle diseases like hypertension and diabetes^27–29^. Hypertension and diabetes might also get triggered by stress and anxiety, which can easily be seen among middle-aged adults in India^30^. Besides, undiagnosed hypertension and diabetes are likely to be more among lower social and economic groups^31^. Frequent substance use from an early age can increase cancer risk. This pattern was confirmed in the present study, as the cancer risk was significantly higher among past tobacco consumers. Physical activity is integral to maintaining good health^32^. However, the higher risk of heart disease or stroke in adults and elderly involved in physical activity found in the present study contradicts that notion. Note that, in the present study, individuals who indulge in either moderate or vigorous activities were considered physically active. High-intensity physical activities possibly increase the risk for cardiac diseases^33^. A study indicated the risk of acute ischemic stroke onset among adults who indulged in sudden rigorous physical activities with a past sedentary lifestyle^34^. Thus, recommended moderate and habitual exercise behaviour for better physical and mental health. Consistent with a prior study, the present study found depression associated with many chronic diseases^35^. Residing in a rural setting increased the risk of developing arthritis among all adults and elderly. This may be due to the larger proportion of adults and elderly of lower economic status in rural areas who cannot afford the high health cost of seeking medical help for joint pains, which thus leads to arthritis in later life^36^. While low economic status was found to be associated with arthritis in rural settings, consistent with the present study, the changing lifestyles due to urbanization and industrialization may increase the risk of other chronic diseases in urban settings^37^.

Contrary to the morbidity theory of developed nations, the present study brings attention to the threat of expansion of morbidity in the Indian context. There is clear evidence that the early onset of chronic diseases has extended the life of individuals living with a morbid condition, especially among independent and working-age populations. Although the prevalence of hypertension is higher among adults and the elderly compared to other chronic diseases, a higher risk of neurological diseases in middle-aged adults (45–54 age group) can bring another burden in the Indian context.

Among all the characteristics, depression emerged as a significant risk of developing chronic diseases, suggesting the need to implement policies that balance the physical and mental health of adults and the elderly in India. The threat of urbanization and industrialization is easily visible from the reduced urban–rural gap of median age of onset of chronic diseases in the 45–54 age group population. This can overburden the rural areas struggling with the higher prevalence of arthritis due to poor medical infrastructure. The higher risk of all chronic diseases among the richest wealth quintile households necessitates focusing on individuals from every sphere without leaving anyone behind.

Despite having the advantage of bringing in light the early onset of chronic diseases and the prognostic risk factors in India, the present study is constrained by a few limitations too. Since biometric information was available only for a few diseases, the present study considered self-reported chronic diseases ever diagnosed by a health professional. The question on onset was asked only to those who self-reported being diagnosed with these conditions. Those who were unaware of their condition but had been diagnosed with a condition using a biomarker were not asked any questions on the onset of the disease. Age of onset measure might be underreported as we know that, in developing countries like India (especially in rural areas), a large proportion of individuals do not receive proper medical attention on time and are left undiagnosed at the actual age of disease origin. Moreover, with the changes in the medical system over the years, like improvement in diagnostic techniques, awareness, facilities and benefits of government health insurance schemes, the comparison across the age groups might be an issue and must be cautioned before any conclusion. Such difficulties were highly associated with the absence of prominent health data till date. However, we acknowledge the increasing burden of chronic diseases in every age group population. Today, chronic diseases are traveling rapidly toward the younger population. Still, we do not have proper evidence to point out the need for policies toward the health of the working-age adults, which covers more than half of India’s population. Thus, we proceeded with the current information of onset, showing its early occurrences and prognostic factors. In accordance to these, here are brief limitations. First, the chronic diseases used are medically diagnosed but self-reported. We acknowledge that a significant proportion of population are un-diagnosed with some of the chronic conditions. Second, the present study did not establish any causality inference and analysed the cross-sectional data due to data limitation. Future research is needed to understand such aspects using longitudinal data. More research on adults and the elderly is required to point out the need for health data and policies toward a healthy and better nation's future.

## Conclusion

The present study adds to the literature by suggesting that chronic diseases are spreading faster among the population and emerging earlier among the younger generation, especially in the independent and working age groups. The findings indicating a higher risk of all seven chronic diseases in the middle-aged adults (45–54 age group) contribute to the debate about whether the younger generation is healthier than its predecessors. The emerging evidence of the early onset of neurological diseases in middle-aged adults (i.e., among the 45–54 age group) reminds us of the need to reinforce a balance between the physical and mental life of individuals. Our findings highlighted the importance of targeting the whole population, i.e., educated and non-educated, urban and rural, rich and poor, as they all struggle with some chronic disease. Notably, the association of depression and substance use with chronic diseases provides the rationale to move away from siloed policies and research conditioned to a few targeted populations. Looking at the sedentary lifestyles of younger generations and in accordance with the findings of the present study, programs are required to promote habitual physical activity. The early onset of chronic diseases in the independent and working-age category (45–54 years) can have many social and economic implications. For instance, it can create a greater healthcare burden when these individuals grow older with these diseases. So, proper interventions and policies are required to tackle such situations in the future. Further, proper health data is needed, so future research may not be obstructed.

## Supplementary Information

Supplementary Information.

## Data Availability

The datasets used for this study are publicly available and can be accessed through: https://www.iipsindia.ac.in/content/LASI-data by filling a data request form along with a valid Identity proof.
