# Supplementary material for: Examining chronic disease onset across varying age groups of Indian adults using competing risk analysis
Source: Sci Rep. 2023 Apr 10;13:5848. doi: 10.1038/s41598-023-32861-5 (PMC10086019; doi:10.1038/s41598-023-32861-5)
Supplement: Supplementary file 1 — Supplementary Information. [file 41598_2023_32861_MOESM1_ESM.pdf]

Appendix

Appendix 1: Description of explanatory variables

S. No.	Explanatory variables	Description
**1.**	Age groups	"45-54 years"; "55-64 years" and "65 years and above"
**2.**	Sex of the individual	Male; Female
**3.**	Educational attainment	Illiterate/None; less than 5 years; 5-9 years; 10 years or more
**4.**	Working status	Currently working; ever worked but not working; never worked
**5.**	Marital status	Married/Live in; Never married/Widowed/Separated/Divorced
**6.**	Living arrangement	Living alone; living with spouse, children, and/or others; living with children and/or others
**7.**	Depression	No; Yes<br>Depression is calculated using the short form of the Composite International Diagnostic Interview (CIDI-SF) scale score, which is a standardized diagnostic interview used in large survey studies. This scale ranges from 0 to 10 and measures major episodic depression among individuals. Prior studies suggest that individuals who endorse five or more symptoms for at least two weeks have 89% probability of meeting the CIDI criteria of major episodic depression, with excellent reliability indicated through Cronbach's alpha (α=0.8). So, the present study coded individuals as depressed (yes) if their score is 5 or more out of 10 symptoms and no otherwise.
**8.**	Tobacco consumption	Never; Past user; Current user
**9.**	Alcohol consumption	Never; Past user; Current user
**10.**	Physical activity	Inactive; Active<br>Physical activity was measured in two forms: moderate activity and vigorous activity. According to WHO recommendation, 75 minutes of vigorous and 150 minutes of moderate physical activity is suitable for adults for being physically active. So, individuals indulged in either moderate or vigorous activity was considered physically active. Otherwise physically inactive if neither of moderate nor vigorous activity present.
**11.**	Caste	Scheduled caste/scheduled tribe; Other Backward class; Others
**12.**	Religion	Hindu; Muslim; Other
**13.**	Place of residence	Rural; Urban
**14.**	Monthly per capita expenditure (MPCE) quintile of household	Poorest; Poorer; Middle; Richer; Richest

Appendix 2: Median age of onset of any chronic disease across age groups of adults and elderly with individual, behavioral and socio-demographic characteristics in India.

Characteristics	Age groups							
	45-54		55-64		65+		All	
	Median	p-value	Median	p-value	Median	p-value	Median	p-value
Individual								
**Sex**								
Male	46	0.143	55	<0.001	65	0.045	55	<0.001
Female	46		54		64		53	
**Education level (in years)**								
No Education	46	<0.001	55	<0.001	66	<0.001	57	<0.001
Less than 5 years	46		55		65		56	
5-9 years completed	46		54		64		52	
10 years or more	45		54		61		51	
**Working Status**								
Currently working	46	0.011	55	<0.001	64	<0.001	51	<0.001
Ever worked but not working	46		55		65		59	
Never worked	45		54		64		54	
**Marital Status**								
Currently married/live in	46	0.003	54	0.004	64	<0.001	52	<0.001
Separated/Divorced/Widowed/Never	46		55		66		59	
**Living Arrangement**								
Living alone	46	0.036	54	0.059	65	<0.001	60	<0.001
Living with spouse, children and/or others	46		54		64		52	
Living with children and/or others	46		55		66		58	
**CIDI-SF Depression status**								
No	46	0.162	55	0.417	64	0.039	54	0.261
Yes	45		54		65		54	
Behavioural								
**Tobacco consumption**								
Never used	46	<0.001	54	<0.001	64	<0.001	53	<0.001
Past user	45		55		65		58	
User	46		55		66		55	
**Alcohol consumption**								
Never used	46	0.059	54	<0.001	64	0.739	54	<0.001
Past user	46		55		65		55	
Current User	46		55		65		53	

**Physical activity status**								
Physically inactive	46	0.014	55	0.420	63	<0.001	52	<0.001
Physically active	46		54		65		54	
Socio-demographic								
**Caste**								
SC/ST	46	<0.001	55	<0.001	65	<0.001	54	0.287
OBC	46		55		65		54	
Others	45		54		64		54	
**Religion**								
Hindu	46	<0.001	55	0.034	64	<0.001	54	<0.001
Muslim	45		54		64		54	
Others	45		54		65		54	
**Place of Residence**								
Rural	46	<0.001	55	<0.001	66	<0.001	55	<0.001
Urban	45		54		63		53	
**MPCE quintile**								
Poorest	46	0.002	55	<0.001	65	0.002	55	<0.001
Poorer	46		55		65		55	
Middle	46		55		65		54	
Richer	46		54		64		54	
Richest	45		54		63		53	
**Total**	**45**		**54**		**64**		**53**	

Note: p value is from log rank test

Appendix 3: Measures of goodness of fit of the Parametric survival regression for the age of onset of seven selected chronic diseases in India.

Hypertension				
**Model**	**Sample (N)**	**Likelihood**	**AIC**	**BIC**
Exponential	65239	-41132.2	82388.3	82951.6
Weibull	65239	-18109.9	36345.8	36918.2
Lognormal	65239	-18198.3	36522.5	37094.9
Loglogistic	65239	-17612.1	35350.3	35922.7

Diabetes				
**Model**	**Sample (N)**	**Likelihood**	**AIC**	**BIC**
Exponential	65236	-24102.8	48329.5	48892.8
Weibull	65236	-14458.6	29043.2	29615.6
Lognormal	65236	-14352.2	28830.4	29402.8
Loglogistic	65236	-14272.9	28671.7	29244.1

Lung Disease				
**Model**	**Sample (N)**	**Likelihood**	**AIC**	**BIC**
Exponential	65246	-13861.1	27846.1	28409.4
Weibull	65246	-10951.2	22028.3	22600.7
Lognormal	65246	-11462.8	23051.6	23624.0
Loglogistic	65246	-10966.8	22059.7	22632.1

Heart Disease & Stroke				
**Model**	**Sample (N)**	**Likelihood**	**AIC**	**BIC**
Exponential	65245	-12271.1	24666.1	25229.5
Weibull	65245	-8456.7	17039.4	17611.8
Lognormal	65245	-8497.2	17120.5	17692.9
Loglogistic	65245	-8426.2	16978.5	17550.9

Arthritis				
**Model**	**Sample (N)**	**Likelihood**	**AIC**	**BIC**
Exponential	65247	-17365.3	34854.7	35418.0
Weibull	65247	-11107.1	22340.3	22912.7
Lognormal	65247	-11145.8	22417.5	22989.9
Loglogistic	65247	-11043.2	22212.4	22784.8

Neurological				
**Model**	**Sample (N)**	**Likelihood**	**AIC**	**BIC**
Exponential	65238	-6022.3	12168.6	12731.9
Weibull	65238	-4889.8	9905.5	10477.9
Lognormal	65238	-5026.7	10179.4	10751.8
Loglogistic	65238	-4891.1	9908.2	10480.6

Cancer				
**Model**	**Sample (N)**	**Likelihood**	**AIC**	**BIC**
Exponential	65245	-2468.2	5060.3	5623.7
Weibull	65245	-2042.1	4210.2	4782.6
Lognormal	65245	-2050.4	4226.9	4799.3
Loglogistic	65245	-2041.8	4209.7	4782.1

Note: The sample size of these chronic diseases is obtained after excluding the missing observations of the respective chronic diseases in total analytical sample of 65,258 individuals

Appendix 4: Accelerated failure time from Loglogistic and Weibull regression model showing association of selected chronic diseases with individual, behavioral and socio-demographic characteristics of adults and elderly in India.

Characteristics	Chronic Diseases													
	Hypertension		Diabetes		Lung Disease		Heart disease & stroke		Arthritis		Neurological		Cancer	
	**TR**	**Confidence Interval**	**TR**	**Confidence Interval**	**TR**	**Confidence Interval**	**TR**	**Confidence Interval**	**TR**	**Confidence Interval**	**TR**	**Confidence Interval**	**TR**	**Confidence Interval**
Individual														
**Age groups**														
65+ (Ref)														
55-64	0.86**	(0.85 - 0.88)	0.85**	(0.82 - 0.87)	0.85**	(0.82 - 0.89)	0.86**	(0.83 - 0.89)	0.86**	(0.84 - 0.88)	0.83**	(0.8 - 0.87)	0.81**	(0.76 - 0.87)
45-54	0.76**	(0.74 - 0.79)	0.77**	(0.73 - 0.83)	0.82**	(0.74 - 0.92)	0.78**	(0.74 - 0.83)	0.77**	(0.73 - 0.81)	0.68**	(0.64 - 0.72)	0.7**	(0.64 - 0.77)
**Individual's Sex**														
Male (Ref)														
Female	0.94**	(0.92 - 0.96)	1	(0.97 - 1.03)	1.03	(0.99 - 1.08)	1.02	(0.99 - 1.06)	0.92**	(0.9 - 0.94)	1	(0.96 - 1.05)	0.91**	(0.84 - 0.99)
**Level of education**														
None (Ref)														
Less than 5 years	0.97**	(0.96 - 0.99)	0.94**	(0.91 - 0.97)	0.98	(0.94 - 1.02)	0.97	(0.94 - 1.02)	0.98**	(0.96 - 1)	1	(0.94 - 1.05)	0.89	(0.77 - 1.04)
5-9 years completed	0.94**	(0.92 - 0.97)	0.92**	(0.88 - 0.97)	0.97	(0.93 - 1.02)	0.95**	(0.92 - 1)	0.97	(0.94 - 1)	0.98	(0.93 - 1.02)	1	(0.92 - 1.08)
10 years or more	0.94**	(0.92 - 0.95)	0.89**	(0.85 - 0.93)	0.99	(0.89 - 1.1)	0.94	(0.88 - 1)	0.97**	(0.93 - 1)	1	(0.94 - 1.06)	1	(0.9 - 1.11)
**Working status**														
Currently working (Ref)														
Ever worked but not working currently	0.97**	(0.95 - 0.99)	0.95**	(0.92 - 0.98)	0.94**	(0.89 - 1)	0.91**	(0.88 - 0.95)	1	(0.97 - 1.04)	0.9**	(0.86 - 0.95)	0.97	(0.91 - 1.04)
Never Worked	0.97**	(0.96 - 0.98)	0.94**	(0.91 - 0.96)	0.95**	(0.91 - 0.99)	0.92**	(0.88 - 0.96)	1.01	(0.99 - 1.03)	0.89**	(0.84 - 0.94)	0.99	(0.92 - 1.08)
**Marital Status**														
Currently married/live in (Ref)														
Separated/Divorced/ Widowed/Never	1.02	(0.99 - 1.05)	1.07**	(1.01 - 1.13)	0.86**	(0.76 - 0.97)	0.92	(0.85 - 1)	0.98	(0.92 - 1.04)	0.88**	(0.79 - 0.99)	0.96	(0.8 - 1.15)
**Living Arrangement**														
Living alone (Ref)														
Living with spouse, children and/or others	0.99	(0.96 - 1.03)	0.98	(0.92 - 1.05)	0.85**	(0.73 - 0.98)	0.85**	(0.77 - 0.93)	0.93**	(0.88 - 1)	0.86**	(0.75 - 0.98)	0.76	(0.56 - 1.03)
Living with children and/or others	0.99	(0.96 - 1.02)	0.98	(0.92 - 1.04)	0.99	(0.9 - 1.09)	0.95	(0.9 - 1.01)	0.98	(0.93 - 1.03)	1.01	(0.93 - 1.08)	0.79**	(0.66 - 0.95)
**CIDI-SF Depression status**														
Not depressed (Ref)														
Depressed	0.94**	(0.91 - 0.97)	0.93	(0.87 - 1)	0.97	(0.93 - 1.02)	0.91**	(0.88 - 0.94)	0.96**	(0.93 - 0.98)	0.85**	(0.81 - 0.9)	0.94	(0.87 - 1.02)
Behavioural														
**Tobacco consumption**														
Never used (Ref)														

Past user	0.99	(0.96 - 1.01)	1.04**	(1 - 1.07)	0.92**	(0.89 - 0.96)	0.89**	(0.84 - 0.94)	1.01	(0.98 - 1.04)	0.92	(0.85 - 1.01)	0.85**	(0.77 - 0.95)
User	1.03**	(1.02 - 1.04)	1.06**	(1.03 - 1.08)	1	(0.97 - 1.03)	1.01	(0.98 - 1.03)	1.01	(0.99 - 1.03)	0.95**	(0.91 - 0.99)	1.01	(0.94 - 1.09)
**Alcohol consumption**														
Never used (Ref)														
Past user	0.99	(0.97 - 1)	0.98	(0.95 - 1.01)	0.95**	(0.92 - 0.99)	0.99	(0.95 - 1.02)	1	(0.97 - 1.02)	0.98	(0.92 - 1.03)	1.04	(0.95 - 1.14)
Current User	1	(0.97 - 1.03)	1.04**	(1.01 - 1.08)	1.04	(0.99 - 1.1)	0.98	(0.88 - 1.08)	1.02	(0.99 - 1.06)	0.99	(0.89 - 1.08)	1.27**	(1.06 - 1.53)
**Physical activity status**														
Physically inactive (Ref)														
Physically active	0.99	(0.97 - 1.02)	1.01	(0.97 - 1.04)	1.06	(0.97 - 1.16)	0.95**	(0.91 - 0.99)	1.01	(0.98 - 1.05)	1.05	(0.98 - 1.13)	0.95	(0.87 - 1.04)
Socio-demographic														
**Caste**														
SC/ST (Ref)														
OBC	1	(0.99 - 1.01)	1	(0.97 - 1.02)	1	(0.96 - 1.04)	1	(0.98 - 1.03)	1	(0.98 - 1.02)	0.96	(0.9 - 1.02)	1.05	(0.95 - 1.16)
Others	1	(0.99 - 1.01)	1	(0.97 - 1.02)	1.05	(0.99 - 1.11)	1.01	(0.98 - 1.04)	1.03	(1 - 1.06)	0.99	(0.92 - 1.07)	0.95	(0.87 - 1.04)
**Religion**														
Hindu (Ref)														
Muslim	0.94**	(0.91 - 0.98)	0.91**	(0.85 - 0.98)	0.97	(0.93 - 1.03)	0.97	(0.93 - 1.01)	1	(0.97 - 1.04)	1.03	(0.97 - 1.1)	1.02	(0.94 - 1.12)
Others	1	(0.97 - 1.03)	1	(0.96 - 1.04)	0.98	(0.92 - 1.05)	0.97	(0.92 - 1.02)	1.04**	(1 - 1.08)	0.95	(0.83 - 1.07)	0.94	(0.85 - 1.03)
**Place of Residence**														
Rural (Ref)														
Urban	0.92**	(0.9 - 0.93)	0.89**	(0.87 - 0.92)	0.99	(0.96 - 1.03)	0.95**	(0.92 - 0.99)	1	(0.98 - 1.03)	0.98	(0.94 - 1.03)	0.94	(0.87 - 1.02)
**MPCE quintile**														
Poorest (Ref)														
Poorer	0.97**	(0.96 - 0.99)	0.98	(0.95 - 1.01)	0.98	(0.95 - 1.02)	0.98	(0.95 - 1.01)	0.97**	(0.95 - 1)	0.97	(0.92 - 1.03)	0.99	(0.88 - 1.11)
Middle	0.97**	(0.95 - 0.98)	0.97**	(0.94 - 1)	0.97	(0.93 - 1.01)	0.97	(0.93 - 1.01)	0.98	(0.95 - 1.02)	0.99	(0.94 - 1.05)	0.99	(0.89 - 1.1)
Richer	0.95**	(0.93 - 0.96)	0.93**	(0.9 - 0.96)	0.97	(0.92 - 1.02)	0.96**	(0.94 - 0.99)	0.96**	(0.94 - 0.99)	0.96	(0.91 - 1.02)	0.88	(0.76 - 1.01)
Richest	0.92**	(0.9 - 0.95)	0.88**	(0.84 - 0.93)	0.89**	(0.8 - 0.98)	0.91**	(0.87 - 0.95)	0.95**	(0.91 - 0.99)	0.98	(0.92 - 1.04)	0.86**	(0.77 - 0.96)

Note: adjusted for individual, behavioral and socio-demographic characteristics; state fixed effect is also considered; ** $p<0.05$; TR=time ratio; Loglogistic accelerated failure time model was fitted for hypertension, diabetes, heart disease/stroke, arthritis, and cancer and the Weibull accelerated failure time model was employed in lung and neurological diseases
